# Quality of life after immune suppressive therapy in aplastic anemia

**DOI:** 10.1007/s00277-024-05731-x

**Published:** 2024-04-05

**Authors:** Iris N. Lommerse, Chris Hinnen, Liesbeth M. van Vliet, Beke Schubert, Jens Panse, Constantijn J. M. Halkes, Jennifer M.-L. Tjon

**Affiliations:** 1 Department of Hematology, Leiden University Medical Centre, Leiden, The Netherlands; 2 Department of Psychology, Leiden University Medical Centre, Leiden, The Netherlands; 3 Health, Medical and Neuropsychology Unit, Institute of Psychology, Leiden University, Leiden, The Netherlands; 4 Department of Hematology, HagaZiekenhuis, The Hague, the Netherlands; 5 Department of Oncology, Hematology, Hemostaseology and Stem Cell Transplantation, University Hospital RWTH Aachen, Aachen, Germany

**Keywords:** Aplastic Anemia, Hematology, Quality of life, Immune Suppressive Treatment

## Abstract

Acquired aplastic anemia (AA) is a rare form of immune-mediated bone marrow failure, which can result in life-threatening infections or bleeding if left untreated. Treatment consists of either immune suppressive therapy (IST) or allogeneic stem cell transplantation (alloHSCT). While considerable research has been published regarding survival, response rate and toxicity of both treatments, knowledge on the impact on quality of life (QoL) is scarce. We used the recently developed AA-specific QoL questionnaire (QLQ-AA/PNH-54) to evaluate QoL in a single center cohort of AA patients who were successfully treated with IST. The 54 questions represent 12 different QoL domains. Results were analyzed for all patients and grouped based on hematologic response (complete response (CR) or partial response (PR)). Thirty-six successfully treated adult patients (15 in CR, 21 in PR) completed the questionnaire (median age 54 years, range 21–71; median time since last IST 5 years, range 0–41). Fatigue was experienced by 83% of patients. Even though total QoL scores did not significantly differ between patients with PR and CR (105 vs 92, p-value 0,17) there appeared to be a trend towards higher scores in patients with PR, especially in domains concerning psychological wellbeing. This trend was most clear in the domains fear of progression (2,12 in PR patients vs 1,73 in CR patients; p-value 0,08) and role functioning (2,22 vs 1,88; p-value 0,07). In conclusion, patients with AA continue to experience psychological and physical effects despite successful IST.

**Supplementary Information:**

The online version contains supplementary material available at 10.1007/s00277-024-05731-x.

## Introduction

Acquired aplastic anemia (AA) is a rare auto-immune mediated disease, characterized by rapidly progressive bone marrow failure resulting in pancytopenia. AA is closely interrelated with paroxysmal nocturnal hemoglobinuria (PNH), a condition in which the acquired loss of glycosyl phosphatidylinositol (GPI)-anchored molecules on the surface of hematopoietic cells leads to complement mediated cell lysis. Treatment of AA consists of either immune suppressive therapy (IST) or allogeneic hematopoietic stem cell transplantation (alloHSCT) [1]. A Cochrane review comparing survival in young patients after IST or alloHSCT concluded there was insufficient proof to state that the two treatment modalities differ in overall survival rates [2]. Nevertheless, both treatment modalities have their own specific (dis)advantages. For instance, successful alloHSCT leads to quick recovery of hematopoiesis, but can lead to transplant-related mortality or impairing (chronic) graft versus host disease (GvHD) in up to 44% of patients [3]. IST as first-line treatment has less direct side effects, but recovery of hematopoiesis usually takes several months and is often partial with persisting (mild) cytopenia. Furthermore, the risk of relapse is 35% and secondary clonal evolution into myeloid malignancies such as myelodysplastic syndrome (MDS) or acute myeloid leukemia (AML) is seen in up to 16% of patients [4, 5]. Currently, both national and international guidelines recommend alloHSCT as first-line treatment for patients under 40 with an available HLA matched sibling donor. For patients over 40 or patients without a suitable donor, IST consisting of horse derived anti thymocyte-globulin (hATG) in combination with ciclosporin (with or without eltrombopag) is the preferred treatment [6–8].

To date, most research on treatment outcome after alloHSCT and IST focuses on survival, toxicity and hematologic recovery. Even though quality of life (QoL) is increasingly recognized as an important treatment outcome, knowledge of QoL after treatment for AA is virtually lacking [9, 10]. Part of the difficulty is that until recently, there was no QoL questionnaire that took into account the disease specific symptoms associated with AA. A few studies tried to approach QoL by using treatment toxicity, drug requirement or GvHD as a proxy-parameter, or used non-disease specific questionnaires like the SF-36 scale or fatigue scores [11–13]. From these studies, we learned that GvHD can be an important factor influencing the QoL in patients after alloHSCT. However, as long term side effects of IST are generally more subtle than GvHD (e.g. persisting mild cytopenias) the approach with proxy-parameters is less suitable to evaluate QoL in patients treated with IST.

Recently, an international research group recognized the need for a questionnaire specifically designed for patients with AA and/or PNH and developed the QLQ-AA/PNH-54 in 2019. The QLQ-AA/PNH-54 has been developed according to EORTC criteria and while it has been psychometrically validated, translated and pre-tested, final field testing including cross-cultural applicability, reliability, and validity is still ongoing [14, 15]. In the present study, a Dutch translation of this questionnaire was used to assess QoL in patients with AA who were successfully treated with IST. The aim of the study was to evaluate use of the questionnaire in daily practice and identification of potential factors influencing Qol. The results from our study may help to better inform patients about the impact of IST on QoL and aid in the shared decision about treatment options.

## Materials and methods

### Patient inclusion

All adult AA patients treated with IST between 1979 and 2020 who still received outpatient follow-up in 2022 at the department of Hematology of the Leiden University Medical Center (LUMC) were asked to participate. IST was defined as a treatment including ciclosporin or anti-thymocyte globulin (ATG) [12]. Patients were excluded if they did not speak Dutch or if they were not in the possession of a valid e-mail address. Since the aim of the study was to investigate QoL after successful IST, patients who did not have hematologic recovery after IST were excluded. Successful treatment was defined as transfusion independence and was further categorized as complete response (CR, normalization of blood counts with a thrombocyte count above 150 × 10^3^/ mm^3^) or partial response (PR, transfusion independent, absolute neutrophil count (ANC) above 0,5 × 10^9^/L, but no normalization of blood counts).

Patients were invited to complete the QoL questionnaire in May 2022 as part of their clinical care evaluation. If patients did not respond to this invitation, a maximum of 2 reminder emails was sent. Patient characteristics and current disease status were obtained from their electronic health record. All data was anonymized before analysis.

### Instrument description and scoring

The AA/PNH specific QoL questionnaire (QLQ-AA/PNH-54) consists of fifty-four multiple choice items (Table 1). The original questionnaire was translated into Dutch to make it suitable for our Dutch population. Backward translation was performed by a native speaker. The official Dutch translation of the QLQ-AA/PNH-54 questionnaire by Niedeggen et al. became available only after performing this analysis but is very similar to the translation used for this study.

**Table 1:** QLQ-AA/PNH-54 questionnaire

During the last 14 days:
1. Have you felt tired?
2. Have you had to rest?
3. Have you been exhausted for days after you exerted yourself?
4. Have you had difficulties getting oud of bed in the morning?
5. Has your body felt heavy?
6. Have you been unable to bring yourself to do things or have you been apathic?
7. Has it been a problem for you to ration your strength?
8. Have you been short of breath?
9. Have you had a tendency to bleed?
10. Have you had problems with susceptibility to infections?
11. Have you had problems with swelling or inflammation of the mouth?
12. Have you had problems sleeping?
13. Has your everyday life been affected by pain?
14. Have you had difficulty standing for an extended period?
15. Has going for a long walk caused you difficulties?
16. Have you had difficulty climbing stairs?
17. Has your normal routine been disrupted?
18. Have your work or other daily activities been restricted?
19. Have you had problems coping with the household chores?
20. Have you had no energy left for your personal life and hobbies?
21. Have you found it a problem to give op sporting activities?
22. Has it bothered you that you were unable to make plans?
23. Has it annoyed you that you had to explain yourself, e.g. why you have been unable to do this or that?
24. Has it bothered you that you were unable to be spontaneous?
25. Has it bothered you that you had to be careful?
26. Have you had to take care all the time to avoid picking up infections?
27. Have you had difficulty concentrating?
28. Has everything revolved around your illness?
29. Has it bothered you repeatedly having to face up to your illness?
30. Have you had signs (e.g. pallor, bruises, dark urine, yellow skin) that repeatedly reminded you of your illness?
31. Has it bothered you to be classified as ill?
32. Has it bothered you that your relatives were upset by your illness?
33. Have you felt supported by friends and family?
34. Have you worried a lot?
35. Has it bothered you that you had to look out for minor symptoms because they could mean something bad?
36. Have you felt good about your body?
37. Have you been bothered by your blood count results?
38. Have you been afraid of a deterioration in your blood count
39. Have you been afraid that therapies might not work?
40. Have you been afraid of a relapse or deterioration?
41. Have you been concerned that there might not be any more therapy for you?
42. Have you felt vulnerable?
43. Have you felt at the mercy of your illness?
44. Have you felt depressed?
45. Did you feel irritable?
46. Has the illness made you feel less attractive?
47. Have you been less interested in sex?
48. Have you been less able to enjoy sex?
49. Have you felt that you were missing out on something in life?
50. Have you been proud of what you achieved despite the illness?
51. Have you been able to do what you wanted?
52. Have you been troubled by thoughts of an uncertain future?
During the last six months:
53. Have you still been able to go on holiday as you wished?
54. Have you missed the interaction with other patients?
Do you have any further comments?

Original QLQ-AA/PNH-54 questionnaire in English as developed by Niedeggen et al. [15]

**Fig. 1:**
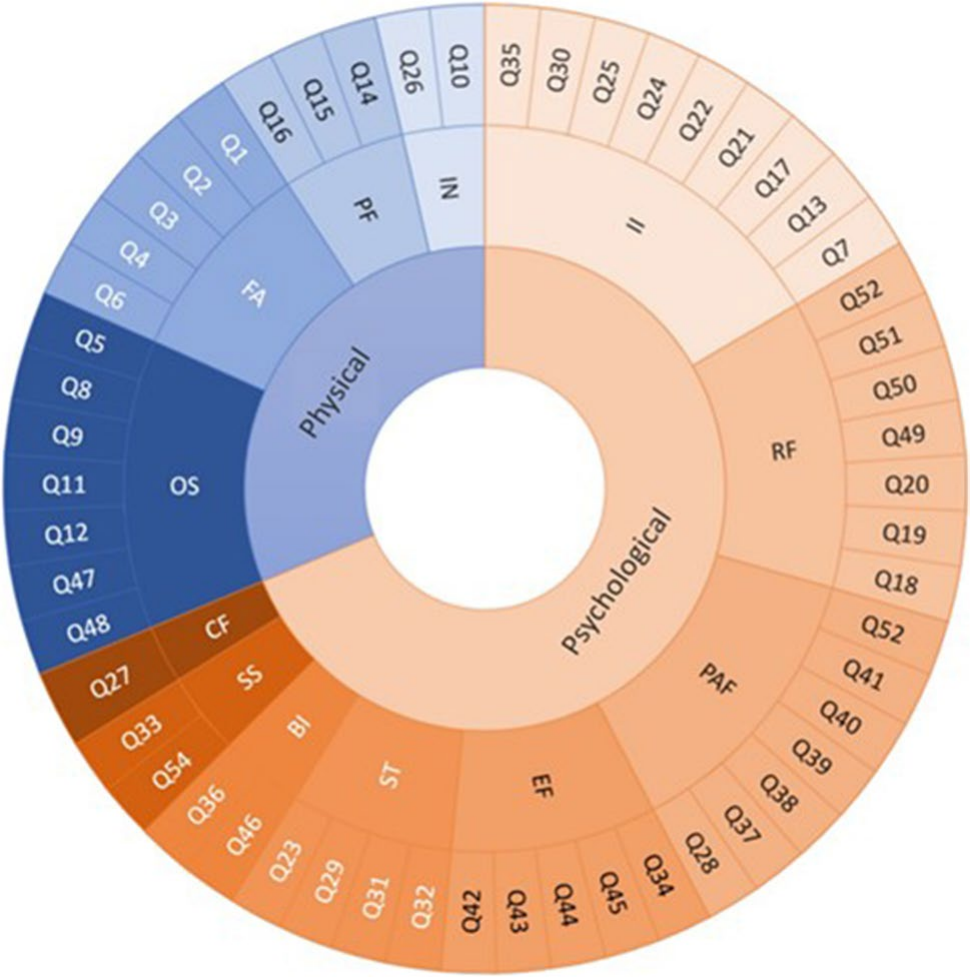
Questions per QoL domain. Questions (outer ring) sorted per domain (middle ring) represented either physical or psychological wellbeing. II: Illness intrusiveness, RF: Role functioning, PAF: Fear of progression, EF: emotional functioning, ST: Stigmatization, BI: Body image, SS: Social support, CF: Cognitive functioning, OS: Other symptoms, FA: Fatigue, PF: Physical functioning, IN: Infections

The questionnaire comprises 12 different domains. Four domains concern physical wellbeing: 5 items assess fatigue (FA), 2 items assess infections (IN), 3 items assess physical functioning (PF) and 7 items assess other symptoms (OS). The other 8 domains concern psychological wellbeing: 2 items assess body image (BI), 1 item assesses cognitive functioning (CF), 5 items assess emotional functioning (EF), 9 items assess illness intrusiveness (II), 7 items assess fear of progression (PAF), 7 items assess role functioning (RF), 2 items assess social support (SS) and 4 items assess stigmatization (ST) (Fig. 1).

All 54 multiple choice questions were scored on a 4 point Likert scale ranging from ‘not at all’ to ‘very often’. Answers were rated from 1 (not at all) to 4 (very often). Fifteen negatively formulated questions were reversely scored. The total score of the QLQ-AA/PNH-54 questionnaire can range between 54 (best QoL) and 216 (worst QoL).

### Statistical analysis and presentation of results

#### Feasibility and validity of questionnaire

We evaluated the feasibility of the questionnaire by observing the mean amount of time needed to fill in the questionnaire. Internal consistency of questions within domains was evaluated by calculating Cronbach’s alpha coefficient. To state that questions are correlated enough to form a domain together, EORTC questionnaire guidelines advise a minimal cut-off value of a Cronbach’s alpha coefficient of 0.7 [16]. We compared our findings to this value and with the Cronbach coefficients presented in the phase 3 study performed by Niedeggen et al. [15].

#### QoL analysis

We explored multiple scoring systems in order to best represent and visualize QoL results. First, the total score per patient was calculated by adding the scores of all answers given. We categorized patients in PR and CR, to evaluate if the attained level of hematologic recovery was of influence on QoL. In addition, we looked into the possible influence on QoL of age, sex, severity of diagnosis, amount of years after IST, thrombocyte count, level of hemoglobin and the presence of a GPI-deficient clone greater than 1%. Linear regression was used to identify patient characteristics influencing QoL on a continuous scale. Independent T-tests were used to compare dichotomous patient characteristics. With a total score consisting of the sum of all questions, domains containing a higher number of questions could influence total score more than domains containing less questions. To avoid this, we calculated the means per domain and subsequently summed these 12 means to produce a weighted total score. We compared total scores and weighted total scores per patient to evaluate if they correlated and thus possibly be interchangeable. We calculated distribution of answers within specific domains and subsequently grouped results based on PR and CR.

## Results

### Patient characteristics

Forty-two adult AA patients treated with IST between 1979 and 2020 with an enduring PR or CR still received outpatient follow up in the LUMC, were Dutch-speaking and in the possession of a functioning e-mail address. Thirty-six patients (87%) filled out the questionnaire; the remaining 6 patients did not respond despite two reminders.

The median age of patients returning the questionnaire was 52 years (range 21–71). Fifteen patients (42%) had a CR after treatment, while 21 (58%) responded with a PR. Patient characteristics were comparable between the two groups, except for the mean thrombocyte count (103 × 10^3^/ mm^3^ in the PR group vs 195 × 10^3^/ mm^3^ in the CR group (Table 2).

**Table 2:** Patient characteristics

	Whole cohort (n = 36)	PR (n = 21)	CR (n = 15)	P-score
Median age in years (range)	54 (21 -71)	48 (30–71)	55 (21–66)	P = 0,500
Female	17 (46%)	9 (43%)	8 (53%)	P = 0,535
Median age at diagnose in years (range)	30 (14–65)	30 (17–65)	30 (14–64)	P = 0,999
Diagnosis classification				
Non-severe AA Severe AA Very severe AA	8 (22%) 20 (56%) 8 (22%)	5 (24%) 10 (48%) 6 (29%)	3 (20%) 10 (67%) 2 (13%)	P = 0,462
Current presence of GPI deficient clone				
> 1% > 40% (with overt hemolysis)	10 (28%) 5 (14%)	7 (33%) 5 (24%)	3 (20%) 0	P = 0,468 P = 0,042
Past IST treatment				
Ciclosporin ATG ATG and ciclosporin ATG, ciclosporin and eltrombopag	2 (6%) 9 (25%) 22 (61%) 3 (8%)	1 (5%) 4 (19%) 15 (71%) 1 (5%)	1 (7%) 5 (33%) 7 (47%) 2 (13%)	P = 0,178
Still on active treatment	2 (6%)	1 (5%)	1 (7%)	P = 0.806
Median amount of years after IST (range)	5 (0 – 41)	5 (1–41)	5 (0–37)	P = 0,499
Current median level of hemoglobin (g/dL) (range)	15 (10 – 16)	13 (10 – 16)	15 (11 – 16)	P = 0,176
Current median thrombocyte count (× 10^3^/ mm^3^) (range)	141 (27–415)	103 (27 – 226)	174 (147 – 415)	P = 0,000

### Questionnaire feasibility and internal consistency

On average, it took patients 9.05 min to complete the digital questionnaire (median: 8.16, range 2.44–23.45). We calculated Cronbach’s alpha coefficient to evaluate internal consistency of questions within domains (table S1). Cronbach’s alpha coefficients were similar to those reported by Niedeggen et al., except in domains ‘Infections’ and ‘Social support’, where we observed very low Cronbach’s alpha coefficients (respectively 0.13 and 0.08, compared to 0.79 and 0.63 reported by Niedeggen et al. [15]).

### Higher age is correlated with lower QoL scores

The mean total score of the questionnaire was 99 (range 63–165). Linear logistic regression showed that age had a weak inverse correlation (R^2^ = 0.112, β = -0.63, p = 0.05) to the mean total score (figure S2). The amount of years after treatment did not appear to influence total scores (figure S3), nor did the current level of hemoglobin or the thrombocyte count (Table 3).

**Table 3:** Linear regression, continuous parameters correlating with total score questionnaire

	Coefficient	R^2^	P-score
Age (y)	-0.63x	0.112	0.05
Years after last IST (y)	-0.19x	0.009	0.52
Hemoglobin level (g/dL)	-1.50x	0.004	0.71
Thrombocyte count (× 10^3^/ mm^3^)	-0.10x	0.065	0.13

Categorizing patients based on hematologic response led to a mean total score of 92 (range 65–142) in patients with CR, versus 105 (range 63–165) in patients with PR (p = 0.17). Men scored a mean total of 95, while women scored 105 (p = 0.33). The presence of a GPI-deficient clone greater than 1% did not seem to influence mean total scores (p = 0,82). The mean of the weighted score (see methods) was 22 (range 14–35). Again, the only significant factor of influence seemed to be patient age (coefficient -0.14x; p = 0.03; r square 0.131). Total score and weighted score were highly correlated within patients with a Pearson correlation of 0.99 (p = 0,00) (supplemental figure S1).

### Psychological wellbeing is affected, even after successful IST

To study the impact of a specific domain on QoL, we evaluated mean scores per domain. The highest mean scores per question were observed in domains ‘Role functioning’ (2,08), ‘Body image’ (1,99), ‘Emotional functioning’ (1,97) and ‘Fear of progression’ (1,96). Comparing scores of patients with PR and patients with CR on these 4 domains, a trend was seen in which patients with PR scored higher than patients with CR, with respective p-values of 0,07, 0,10, 0,14 and 0,08. The lowest mean score per question (1,40) for all patients was seen in the domain ‘Infections’ (Table 4).

**Table 4:** Mean QoL scores per domain stratified by hematologic response to treatment

Domain	Whole cohort (n = 36)	PR (n = 21)	CR (n = 15)	p-score
Role functioning	2.08 (SD 0.57)	2.22 (SD 0.57)	1.88 (SD 0.53)	0.07
Body image	1.99 (SD 0.78)	2.17 (SD 0.86)	1.73 (SD 0.59)	0.10
Emotional functioning	1.97 (SD 0.70)	2.11 (SD 0.75)	1.76 (SD 0.58)	0.14
Fear of progression	1.96 (SD 0.69)	2.12 (SD 0.78)	1.73 (SD 0.46)	0.08
Fatigue	1.93 (SD 0.68)	2.02 (SD 0.72)	1.81 (SD 0.63)	0.38
Cognitive functioning	1.83 (SD 0.61)	1.86 (SD 0.48)	1.80 (SD 0.77)	0.80
Illness intrusiveness	1.79 (SD 0.59)	1.92 (SD 0.63)	1.61 (SD 0.50)	0.14
Stigmatization	1.78 (SD 0.75)	1.95 (SD 0.81)	1.53 (SD 0.61)	0.10
Physical functioning	1.76 (SD 0.82)	1.71 (SD 0.70)	1.82 (SD 0.98)	0.70
Social support	1.69 (SD 0.50)	1.64 (SD 0.43)	1.77 (SD 0.59)	0.47
Other symptoms	1.61 (SD 0.45)	1.61 (SD 0.39)	1.62 (SD 0.54)	0.93
Infections	1.40 (SD 0.43)	1.38 (SD 0.47)	1.43 (SD 0.37)	0.72

Mean scores per question per domain in all patients and categorized per hematologic response. PR: Partial response, CR: Complete response, SD: Standard deviation

**Fig. 2:**
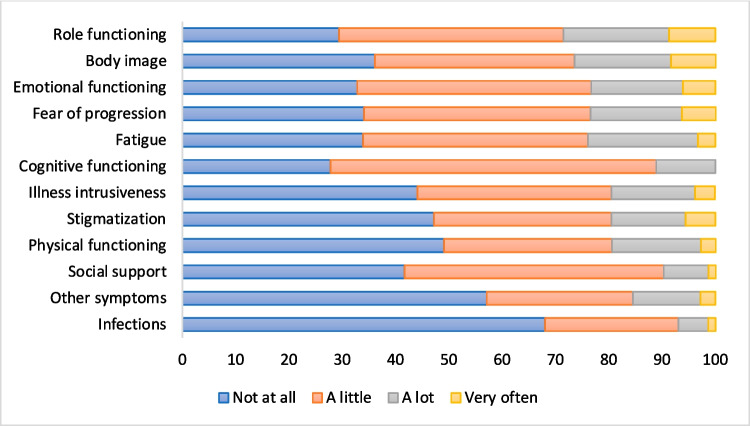
Frequency of chosen answer options per domain (%)

To further zoom in on the actual answers that were given within a domain, we calculated the distribution of answer options for each domain separately (Fig. 2). The answer option ‘not at all’ was chosen most frequently within the physical domains ‘Infections’, ‘Other symptoms’ and ‘Physical functioning’ (respectively 68%, 57% and 49%). In domain ‘Fatigue’ the percentage of questions answered with ‘not at all’ was with 34% relatively low in comparison to the other physical related domains. The one question addressing tiredness specifically, was scored to be relevant (a little, a lot, or very often) for 83% of participants. Questions were scored with ‘a lot’ or ‘very often’ most frequently within the psychological domains ‘Role functioning’ and ‘Body image’ (respectively 29% and 26%).

**Fig. 3:**
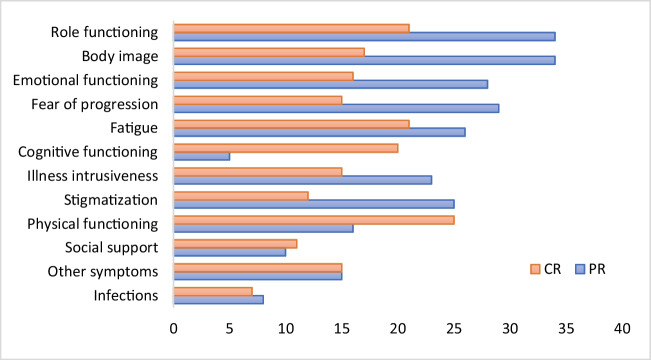
Frequency of chosen answer options ‘a lot’ or ‘very often’ per domain (%), categorized by type of hematologic response

When stratified according to treatment response, patients with PR scored answer options ‘a lot’ or ‘very often’ more frequently than patients with CR on 8 out of 12 domains. Exceptions were ‘Social support’, Physical functioning’, ‘Cognitive functioning’ and ‘Other symptoms’ (Fig. 3). The differences between CR and PR patients was most clear in the psychological domains. In one specific question addressing fear of worsening blood counts, the answer option ‘very often’ was chosen by almost a quarter (24%) of PR patients, whereas in the CR group only 7% of patients gave this same answer.

## Discussion

We evaluated the use of the recently developed AA/PNH-adapted QoL questionnaire (QLQ-AA/PNH-54) in a single center cohort of AA patients after successful IST. This is to date the only disease specific QoL questionnaire targeting patients with AA or PNH [17]. This questionnaire addresses implications of the disease on physical, psychological and social functioning to get insight in overall QoL of AA or PNH patients. In current published literature only one case-report reports using the QLQ-AA/PNH-54 questionnaire to evaluate QoL of a PNH patient before and after treatment [18]. Given the low incidence of AA, inclusion of sufficient patient numbers can be challenging. Our study is the first in which a relatively large group of 36 AA patients is evaluated with the QLQ-AA/PNH-54 questionnaire. Our study shows that AA patients who have been treated with IST can easily use the QLQ-AA/PNH-54 as it took most patients less than 10 min to answer all 54 questions. The medical need to address QoL in AA patients was underlined by the willingness of our AA patients to cooperate, resulting in an high response rate of 87% [19].

The phase 3 study of the QLQ-AA/PNH-54 questionnaire showed robust relevancy, validity and internal consistency and was published in 2019 [15]. Further psychometric validation is pending with an anticipated phase 4 study, which will be performed within the International PNH Interest Group (IPIG) registry. In order to contribute to further validation of the questionnaire, we evaluated the internal consistency of questions within domains by calculating Cronbach’s alpha coefficients. In our cohort, this internal consistency of domains was comparable to that reported by Niedeggen et al.[15], except in ‘Social support’ and ‘Infections’, where a lower Cronbach’s alpha was observed in our cohort. A possible explanation for the difference in Cronbach’s alpha could be the different make-up of cohorts. The cohort used by Niedeggen et al. consisted of both AA and PNH patients, whereas our cohort contained exclusively AA patients. Based on these results it would be advisable to review questions within specific domains for the use of this questionnaire specifically in patients with AA [16].

With ongoing psychometric validation, a uniform scoring algorithm has yet to be developed. For this reason we explored different ways to visualize the results of the QLQ-AA/PNH-54 questionnaire. We first analyzed correlations of different parameters on the total sum score. However, total sum scores entail the risk to overrepresent domains consisting of a higher number of questions. For this reason, in questionnaires like the QLQ-C30 and the Nottingham Health Profile no total sum score is calculated. Instead of using the total sum score, these questionnaires use a weighted sum score that assumes every domain is of equal importance on QoL. Hinz et al. argument against this theory by stating that domains that are more relevant usually also contain more questions, which would justify a greater contribution of these domains to the total score [20]. Ideally one would ask every patient to grade the importance of each domain on his/her QoL, and calculate an individually weighted QoL score. However, this ‘weighing’ of domains can be quite complicated and taxing for patients. We evaluated both the total sum score and the weighted score for our patients and found that both scores led to interchangeable results within patients, with a Pearson correlation of 0.99. The distribution of answer options per domain gave a more visual representation of impact of the 12 domains on QoL and the influence of hematologic response to IST (PR versus CR). We observed different distributions among the PR and CR group. However, to avoid multiple testing errors in small subgroups, we did not perform statistical analysis on these findings.

This first cross-sectional study gives us relevant insights in the QoL of AA patients treated with IST. However, we cannot directly compare QoL scores of our AA population with QoL scores of the general population because the QLQ-AA/PNH-54 is a disease-specific questionnaire. Interestingly, in our cohort higher age seems to be correlated with a better QoL, whereas in studies evaluating QoL in a general population an opposite effect of age on QoL is seen. [21, 22] A possible explanation for this difference is that older AA patients may have different expectations from life than younger patients. For instance, it could be easier for the older patient to adapt to physical limitations caused by disease, because expectations on physical functioning usually decrease with increasing age. On the other hand, younger patients might feel more pressure from society on their performance, and thus feel a greater psychological burden if they fail to meet the standards. In addition to this age-related difference in QoL, our study also shows that fatigue and psychological issues appear to have most impact on QoL, especially in patients with PR. A possible explanation for the psychological burden might be the continuous present risk of progression or clonal cell expansion after IST. The annual check-up could be an ongoing reminder of this, and thus cause a repeating stress factor for patients. This hypothesis might also explain why especially psychological domains of QoL appear to be affected more in patients with a PR than a CR: fluctuating levels of blood count and the worry regarding progression might be particularly high in patients without normalization of blood counts. Affirmation of abnormal blood counts by these yearly blood results could also cause patients to ‘feel’ more ill, even though a slightly lower thrombocyte count or ANC usually does not have a direct effect on physical functioning as it is.

In conclusion, our study shows that the QLQ-AA/PNH-54 is feasible to evaluate QoL in AA patients treated with IST. For the individual patient, the questionnaire can be helpful to open conversation regarding QoL between doctor and patient. Results of the questionnaire can lead to targeted support or interventions, either by the treating physician or a specialized third party. Incorporation of the QoL questionnaire would thus be a valuable addition to the standard follow up and treatment evaluation of AA patients.

### Supplementary Information

Below is the link to the electronic supplementary material.

Supplementary file1 (DOCX 30 KB)

Supplementary file2 (DOCX 34 KB)

Supplementary file3 (DOCX 27 KB)

Supplementary file4 (DOCX 15 KB)
