# Supplementary figures and images for: Quality of life after immune suppressive therapy in aplastic anemia
Source: Ann Hematol. 2024 Apr 5;103(6):2113–21. doi: 10.1007/s00277-024-05731-x (PMC11090919; doi:10.1007/s00277-024-05731-x)

**Fig S1: Correlation between weighted score and total score**


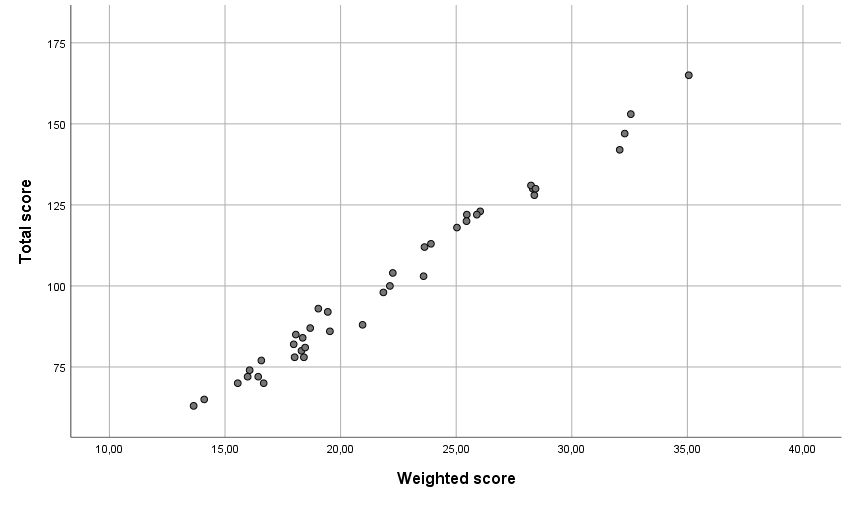

Supplement: Supplementary file 1 — Supplementary file1 (DOCX 30 KB) [file 277_2024_5731_MOESM1_ESM.docx]

**Fig S2: Correlation between age and total score**


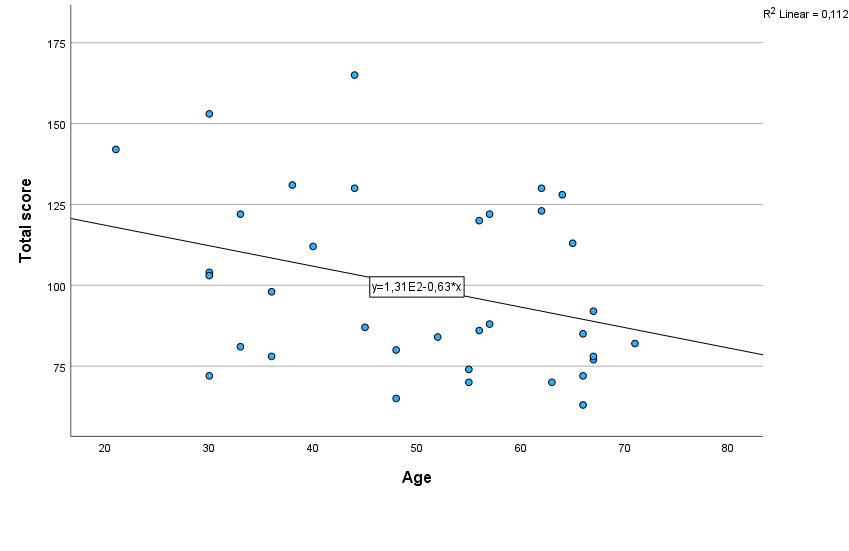

Supplement: Supplementary file 2 — Supplementary file2 (DOCX 34 KB) [file 277_2024_5731_MOESM2_ESM.docx]

**Fig S3: Correlation between time after treatment (years) and total score**


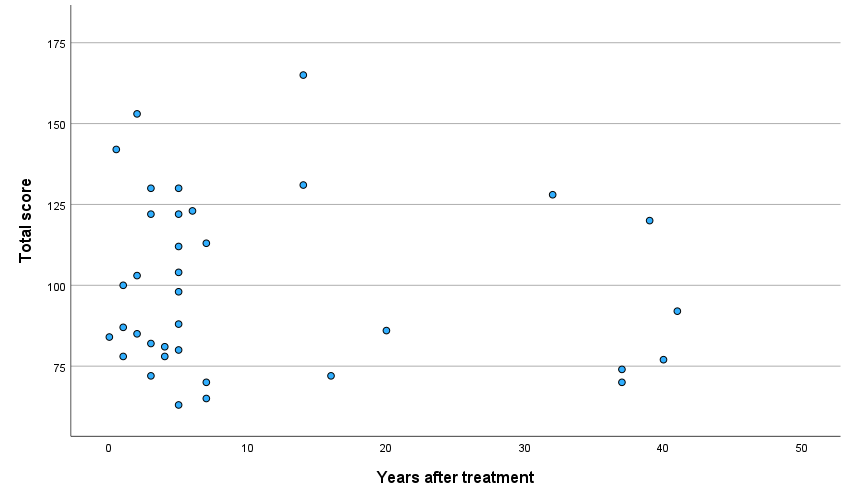

Supplement: Supplementary file 3 — Supplementary file3 (DOCX 27 KB) [file 277_2024_5731_MOESM3_ESM.docx]
