# Supplementary material for: Quality of life after immune suppressive therapy in aplastic anemia
Source: Ann Hematol. 2024 Apr 5;103(6):2113–21. doi: 10.1007/s00277-024-05731-x (PMC11090919; doi:10.1007/s00277-024-05731-x)
Supplement: Supplementary file 4 — Supplementary file4 (DOCX 15 KB) [file 277_2024_5731_MOESM4_ESM.docx]

**Table S1** Internal consistency within domains evaluated using Cronbach’s alpha coefficient

| Domain | Cronbach’s alpha coefficient | Cronbach’s alpha coefficient as reported by Niedeggen et al [15]. |
| --- | --- | --- |
| Fatigue | 0,91 | 0,88 |
| Other symptoms | 0,71 | 0,75 |
| Illness intrusiveness | 0,88 | 0,92 |
| Infections | 0,13 | 0,79 |
| Stigmatization | 0,86 | 0,78 |
| Fear of progression | 0,91 | 0,92 |
| Emotional functioning | 0,87 | 0,81 |
| Role functioning | 0,78 | 0,82 |
| Social support | 0,08 | 0,63 |
| Physical functioning | 0,89 | 0,89 |
| Body image | 0,72 | 0,82 |
